# Initial Experience in Single-Incision Transumbilical Laparoscopic Liver Resection: Indications, Potential Benefits, and Limitations

**DOI:** 10.1155/2012/921973

**Published:** 2012-09-25

**Authors:** Giovanni Dapri, Livia DiMarco, Guy-Bernard Cadière, Vincent Donckier

**Affiliations:** ^1^Department of Gastrointestinal Surgery, European School of Laparoscopic Surgery, Saint-Pierre University Hospital, 1000 Brussels, Belgium; ^2^Department of Anesthesiology, Saint-Pierre University Hospital, 1000 Brussels, Belgium; ^3^Liver Unit, Department of Abdominal Surgery, Hôpital Erasme, Université Libre de Bruxelles, 808 Route de Lennik, 1070 Brussels, Belgium

## Abstract

*Background*. Single-incision transumbilical laparoscopic liver resection (SITLLR) has been recently described in limited series. We report our experience in SITLLR and discuss the future of this approach in terms of indications, potential benefits, and limitations, with a special reference to laparoscopic liver resection (LLR). *Patients and Methods*. Six patients underwent SITLLR. Indications were biliary cysts (3 cases), hydatid cysts (2), and colorectal liver metastasis (1). Procedures consisted in cysts unroofing, left lateral lobectomy, pericystectomy, and wedge resection. SITLLR was performed with 11 mm reusable trocar, 10 or 5 mm 30° scopes, 10 mm ultrasound probe, curved reusable instruments, and straight disposable bipolar shears. *Results*. Neither conversion to open surgery nor insertion of supplementary trocars was necessary. Median laparoscopic time was 105.5 minutes and median blood loss 275 mL. Median final umbilical scar length was 1.5 cm, and median length of stay was 4 days. No early or late complications occurred. *Conclusion*. SITLLR remains a challenging procedure. It is feasible in highly selected patients, requiring experience in hepatobiliary and laparoscopic surgery and skills in single-incision laparoscopy. Apart from cosmetic benefit, our experience and literature review did not show significant advantages if compared with multiport LLR, underlying that specific indications remain to be established.

## 1. Introduction

Since the first reports in the nineteen's [1, 2], laparoscopic liver resection (LLR) has now become a well-recognized and accepted procedure for treatment of liver tumors in selected cases. Currently, feasibility, safety, and clinical benefits of LLR have been clearly demonstrated for treatment of both benign [3] and malignant liver tumors [4]. Initially, LLR have been reserved to small lesions, located in anterior liver segments, at distance of major vascular and biliary structures, but, now, the feasibility and safety of LLR for tumors located posteriorly, centrally or requiring a major hepatic resection have been established [5]. A step forward, laparoscopic living donor hepatectomy, including left lobectomy for liver transplantation in children [6] and right hepatectomy for adult liver transplantation, has been proposed by specialized groups [7]. Single-incision transumbilical laparoscopy (SITL), firstly performed in 1992 [8], recently gained interest in general surgery. SITL represents the latest advance of the laparoscopic approach, aiming mainly to improve the cosmetic outcomes, while other potential advantages such as reduced postoperative pain, minimized operative trauma, and reduced hospital stay still need further investigations. SITL has been successfully reported for several abdominal interventions, including appendectomy, cholecystectomy, inguinal and ventral hernia, splenectomy, partial gastrectomy, and colectomy [9].

On the ground of both, the advances of multiport LLR techniques and the recent development of SITL, some surgeons reported the feasibility of single-incision transumbilical laparoscopic liver resection (SITLLR). Most reports concern clinical cases [10–20], while only few centers described series with more than 5 cases [21–28] (Table 1). Accordingly, the feasibility and beyond the potential advantages and disadvantages of this technique remain to be determined. We report herein our initial experience describing 6 patients who underwent SITLLR for benign and malignant liver lesions. We discuss the feasibility of this approach and its potential benefits and limitations, particularly compared with multiport LLR.

## 2. Patients and Methods

Between April 2010 and February 2012, 6 patients were submitted to SITLLR. Patients' characteristics and type of disease are represented in Table 2. The first 5 patients had no previous surgical history, whereas the patient 6 had a laparoscopic total mesorectal excision for a rectal cancer 6 months before. Surgical procedures consisted in biliary cysts unroofing (patients 1, 2, 3), left lateral lobectomy (patient 4), pericystectomy of segment 7 (patient 5), and wedge resection of segment 8 (patient 6). Preoperative work-up was performed by standard hematological and biochemical laboratory evaluations, including relevant tumor markers. All patients had preoperative liver imaging, using CT scan and/or MRI. Additionally, patient 6 had a whole body FDG-PET scan to exclude the presence of extrahepatic metastases.

### 2.1. Surgical Technique

**Figure 1:**
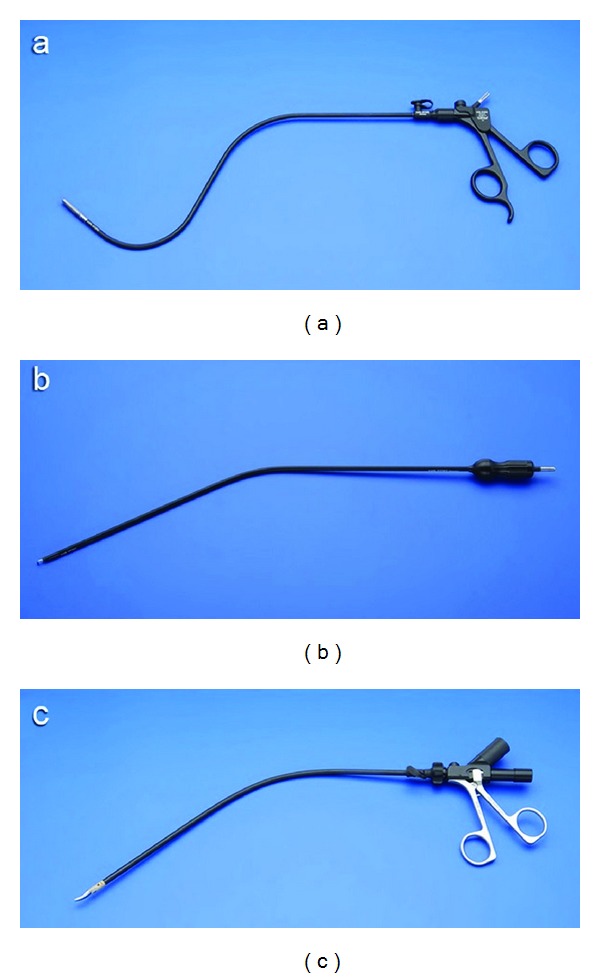
Curved reusable instruments according to DAPRI: grasping forceps III (a), coagulating hook (b), bipolar scissors (c) (courtesy of Karl Storz-Endoskope, Tuttlingen, Germany).

The patient is placed under general anaesthesia in supine position and with the legs apart. The surgeon stands between the patient's legs and the camera-assistant to the patient's left. The original umbilical scar is incised and the fascia opened at 1 cm. A purse-string suture using 1 polydioxanone (PDS) is placed in the fascia, and an 11 mm reusable trocar is inserted inside. A 10 mm 30°, rigid and normal length scope (Karl-Storz Endoskope, Tuttlingen, Germany) is used. A curved reusable grasper (Karl-Storz Endoskope, Tuttlingen, Germany) (Figure 1(a)) is inserted at 10 o'clock position through a separate fascia opening outside the purse-string suture and without trocar. This instrument is maintained in the surgeon's nondominant hand, and it is never changed during the entire procedure. 

#### 2.1.1. Cyst Unroofing

**Figure 2:**
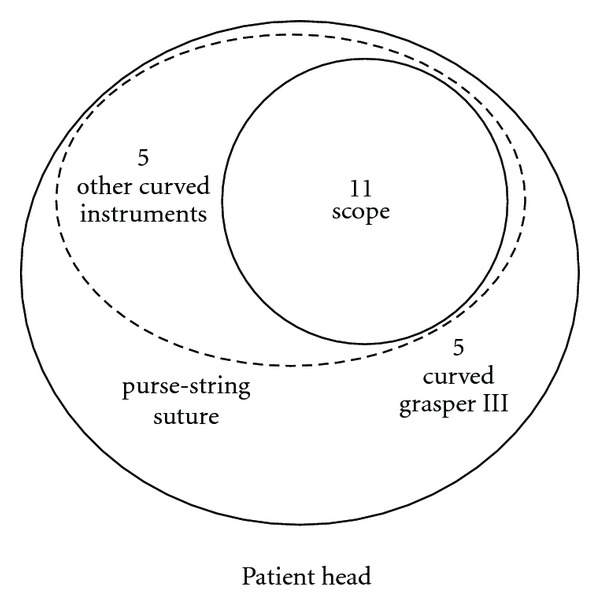
Umbilical access during cyst unroofing: placement of purse-string suture, 11 mm trocar for the 10 mm scope, and 5 mm curved instruments without trocars.

**Figure 3:**
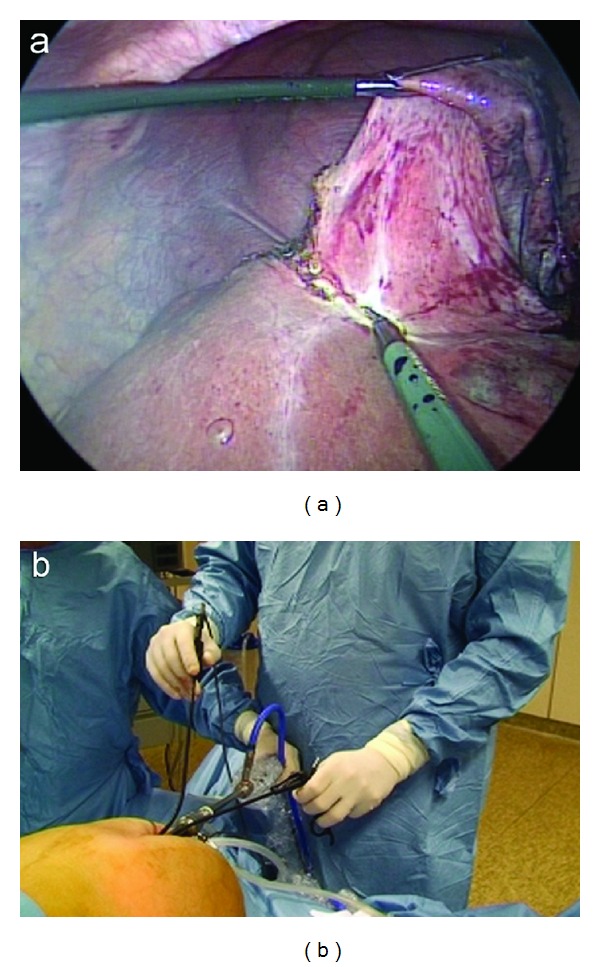
Cyst unroofing: internal working triangulation (a) and external ergonomy (b) using curved reusable instruments.

The other instruments for the surgeon's dominant hand, like the curved reusable coagulating hook (Figure 1(b)), the curved reusable bipolar scissors (Figure 1(c)), and the curved reusable suction device, are changed during the different steps of SITLLR and inserted at 3 o'clock position inside the purse-string suture and besides the 11 mm trocar (Figure 2). The procedure starts with the exploration of the abdominal cavity and identification of the biliary cysts. The cystic domes are identified and incised enough to empty the cystic cavity. Thanks to this manoeuvre a nonconnection between the liver cysts and the biliary tree is evidenced. A meticulously complete excision of the cystic roof is performed. Thanks to the curves of the instruments, the classic working triangulation of laparoscopy is established inside the abdomen (Figure 3(a)), and surgeon is able to work in ergonomic position during the entire procedure (Figure 3(b)). The liver cyst cavities are finally checked for bleeding and left opened without omental patch.

#### 2.1.2. Other SITLLR

**Figure 4:**
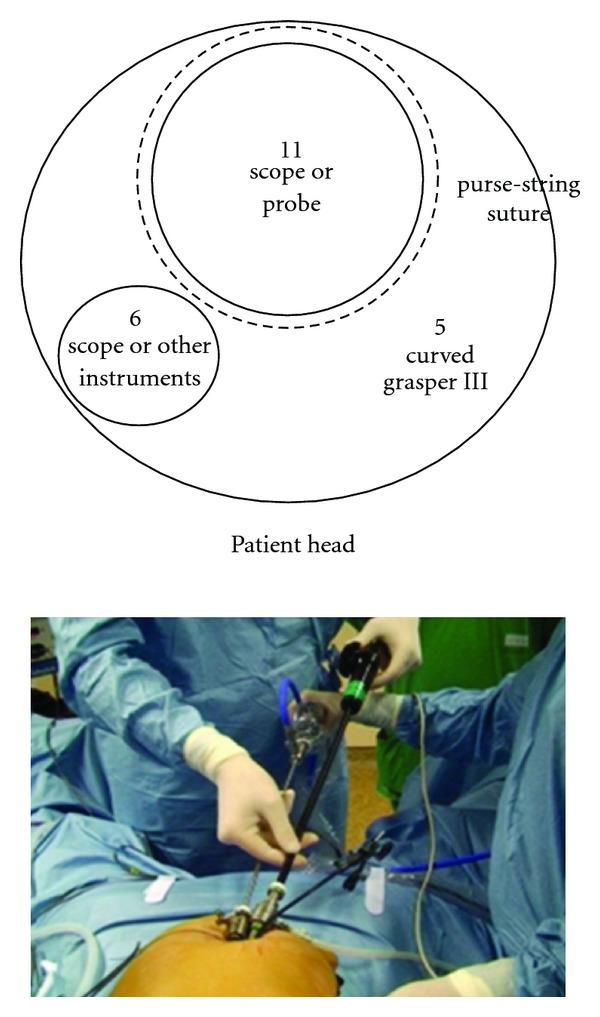
Umbilical access during other SITLLR: IOUS performed with the insertion of the ultrasound probe into the 11 mm trocar and use of long scope through the 6 mm trocar.

**Figure 5:**
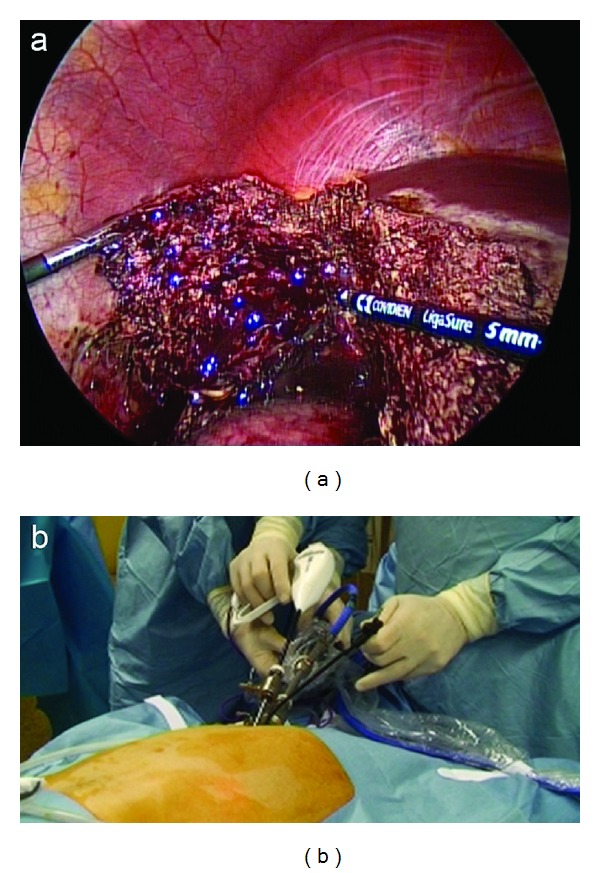
Other SITLLR: internal triangulation using straight disposable bipolar shears and curved reusable grasper (a), and external view (b).

A 6 mm reusable trocar is inserted at 2 o'clock position outside the purse-string suture, in order to accommodate a 5 mm 30°, rigid and longer scope. The flexible laparoscopic multifrequency linear probe is inserted in the 11 mm trocar (Figure 4), and the procedure starts with the exploration of the liver parenchyma through the intraoperative ultrasonography (IOUS), which permits to determine the transection line of the liver parenchyma. Then, the optical system is switched again into a 10 mm scope. A disposable straight bipolar shear (Ligasure V, New Haven, Covidien, CT, US) is inserted through the 6 mm trocar. The liver parenchyma is transected. An internal working triangulation is often created thanks to the curves of the grasper (Figure 5(a)), but an external conflict between the optical system and the handle of the straight bipolar shears is frequently evidenced (Figure 5(b)). If necessary, a straight 5 mm clip applier (Weck Hem-o-lock, Teleflex Medical, Sint-Stevens Woluwe, Belgium) is inserted through the 6 mm trocar.

#### 2.1.3. End of the Procedure

A custom-made plastic bag is introduced in the abdomen through the 11 mm trocar, and the specimen is extracted transumbilically. The instruments and trocars are removed; the umbilical fascia is closed using absorbable sutures, taking care to close the separate openings for the curved grasper and for the 6 mm trocar.

## 3. Results

Neither conversion to open surgery nor insertion of supplementary trocars was necessary. Median total operative time (between skin incision and closure of the fascia) was 126 minutes (range: 89 to 185 min), and median laparoscopic time (between beginning of pneumoperitoneum and removal of the instruments and trocars) was 105.5 minutes (range: 71 to 160 min) (Table 3). Median total blood loss was 275 mL (range: 40 to 500 mL). No intraoperative complications occurred, excepting a major bleeding during the hydatid cyst pericystectomy (patient 5). Median final umbilical scar length was 15 mm (range: 14 to 20 mm). The patients' pain medication was kept low. No early complications were registered within the first postoperative month, and patients were discharged from the hospital between the postoperative day 3 and 5. Pathological evaluation confirmed the preoperative diagnoses of benign biliary cysts, the hydatid liver cysts, and colorectal liver metastasis; for this latter patient the margin of resection was 1 mm. After a median followup of 8 months (range: 3 to 25 months), no late complications related to recurrent disease or to the access-site were observed.

## 4. Discussion

The development of new techniques to reduce the surgical trauma and to minimize the abdominal wall damage is an obvious trend in liver surgery. Accordingly, LLR has been increasingly performed this last decade, becoming now a standardized procedure in selected cases, able to provide significant benefits as compared with classical open liver resections [29, 30]. SITLLR represents an ultimate evolution of the laparoscopic approach to the liver, being considered as very minimally invasive. The objective of SITLLR, beyond the cosmetic gain, is to further reduce the global surgical stress, potentially having a favorable impact on the postoperative evolution. At this point of the experience, several questions on SITLLR remain to be addressed, concerning the feasibility and mostly the reproducibility of this technique, the indications, selection criteria, limitations, effect on postoperative outcomes, and long-term results. It is on these bases that it would be possible to evaluate if SITLLR could become, more than just a technical challenge, a real therapeutic option to improve the outcomes of patients submitted to liver resections. For such evaluation, the growing experience of SITLLR should be closely confronted to the well-established results of LLR, serving as standard of comparison. Several reports have reported the feasibility of SITLLR in selected cases [21–28]. Accordingly [21, 23, 25, 26, 28], our series confirms as SITLLR can be performed without conversion to open surgery or insertion of supplementary trocars, including also the resection of the lesions located in posterior liver segments. From a technical point of view, the objective during SITLLR is to maintain the procedure as similar as possible to the principles of multiport LLR. As a matter of fact, the technique described here is basically close to multiport laparoscopy, with the main difference that it is performed through the same umbilical incision, using instruments close to each other. One of the main rules of laparoscopy, which consists in maintaining the optical system as the bisector of the working triangulation [31], is respected during SITL because the 10 mm scope is maintained in the center of the umbilical access and the instrument for the surgeon's nondominant hand (curved grasper) on the right side of the access, whereas the instruments for the surgeon's dominant hand (coagulating hook, bipolar scissors, bipolar shears, clip applier, suction device) on the left side. The curved grasper is never changed during the entire procedure, whereas the instruments for the surgeon's dominant hand are continuously changed and replaced by the 5 mm scope during the step of IOUS. This step is the only one where one of the above laparoscopic rules is not respected, because the optical system is inserted laterally to give the place to accommodate the ultrasound probe. The technique described here, differently from the common SITL [9], did not increase the cost of standard laparoscopy, because all of the material implemented is reusable, except for the straight bipolar shears used during the parenchymal transection. As during multiport LLR, SITLLR parenchymal transection can be performed using several devices like Harmonic ACE (Ethicon Endosurgery, Cincinnati, OH, US) [21, 26, 28], saline-linked sealing dissector (SH2.0, TissueLink Medical's Dover, NH, US) (12), SonoSurg (Olympus, Tokyo, Japan) [14, 23], cavitron ultrasonic surgical aspirator (CUSA Excel, Valley Lab Inc., Boulder, CO, US) [24], Surgiwand (Covidien, Mansfield, MA, US) [24], Maryland type forceps and bipolar forceps combined with suction irrigation system [25], or Ligasure (Covidien New Haven, CT, US) [26, 28]. We adopted the use of curved coagulating hook and curved bipolar scissors for cystic dome resection and straight bipolar shears for parenchyma transection. Comparing these two instruments, surgeon was immediately confronted with the problem of external conflict between the optical system and the handle of the straight bipolar shears because, differently from the curved tools, both instruments are supported by a straight shaft (Figures 4 and 5). A significant gap was observed between the total and the laparoscopic times. This interval time can be explained by the need to get access to the peritoneal cavity as well as the time to meticulously close the umbilical access and the separate fascial openings at the end of the procedure, while laparoscopic time in this series remained similar to that previously reported [21]. This could be surely improved thanks to the surgeon's learning curve, and the time of laparoscopy could be reduced if particular devices with multiple functions like tissue division, hemostasis, irrigation, and suction are adopted [25]. Blood loss during SITLLR as well as during multiport LLR remains a factor related to the use of specific instruments for the parenchymal transection, to the extension of the parenchyma resected, to the time of transection, and, lastly, to the occurrence of intraoperative complications [21–25, 28]. In one case of this series (patient 5), a pericystectomy performed in segment 7 for a hydatid cyst, a significant bleeding was achieved, most probably in relation to the difficult access to superior and posterior liver segments and to the longer time needed for parenchymal transection. Still, no patients of the series necessitated blood transfusions, and no postoperative early complications were recorded. 

After a median followup of 8 months, we did not achieve any complications related to the access site, to the remaining liver tissue, and to the general patient's conditions, but a longer followup is necessary for the evaluation of recurrent disease and for the appearance of incisional hernia at the access site. Thanks to the technique described here, the umbilical incision length is kept minimal. This result can be obtained because disposable port devices, requiring larger incision [9], are not used. Moreover, tumor's size and pathology are factors influencing the final scar length because malignant tumors superior to 30 mm of diameter need enlargement of the scar for extraction and intact specimen for pathological examination [14, 23, 26]. In our experience benign lesions, like biliary and hydatid cysts, were fragmented at the level of the umbilicus in a plastic bag, while malignant lesions were selected for SITLLR if small and with a diameter inferior to 3 cm [21, 22, 25]. In case of larger lesions, the final scar length has to be enlarged [23, 24, 26], sharing then an increased risk for development of incisional hernia [32, 33]. Such risk can also be associated to the placement of the final drain through the scar [10, 23]. As previously reported [21, 26], and similarly to our attitude during multiport LLR, we did not use a drain. In cases where a drain has to be left in the abdomen, we prefer to use a different abdominal wall punction, out of the umbilicus, in order to avoid the risk of incisional hernia. This punction can also be used during SITLLR to insert a needlescopic (3 mm) instrument [22] or a classic additional trocar [14, 23]. Hence, SITLLR becomes a technique of reduced port surgery, using two accesses, one at the umbilicus and one in another abdominal quadrant. Similarly, a supplementary instrument can also be inserted for technical problems due to tissue manipulation or compromised visualization [22] and noncontrolled perioperative bleeding or limited length of instruments [24]. Other factors of selection for SITLLR are the patient's body mass index and the patient's height, because it can be a cause of conversion to multiport laparoscopy [22, 24]. According to other authors [23, 26], we did not consider previous abdominal surgery, like in our patient 6, as a contraindication to SITLLR. Potential indications for LLR have now been clearly identified, showing essentially no predefined contraindications as compared with open liver resection in terms of disease pathology, including both benign and malignant tumors, either primary hepatocellular carcinoma or liver metastases. Importantly, with the reserves due to the absence of randomized trial and many selection biases, no oncological disadvantages have been shown for laparoscopic approach as compared with standard open procedure, neither in terms of radicality of resection nor for the risk of tumor cell seeding and long-term outcomes [34–38]. At this point of the experience, there are no reasons to consider that the same would be true for SITLLR. From surgeon's side, several statements clearly underlined that LLR should be performed by surgeons experienced in liver resections, meaning knowledge of hepatic anatomy, experience in open liver surgery, and skills in the use of IOUS [30]. Additionally, extensive experience in laparoscopic surgery and ability to identify and control major vascular and biliary structures laparoscopically are mandatory before embarking on multiport LLR [34]. Regarding the application of SITL to the liver, it is most probably reasonable to go back to the initial development of LLR, both in terms of indications and surgeon's experience. Accordingly, small anterior tumors, including malignant lesions, could be selected for SITL if the transection plane is well defined and at distance of the major biliary and vascular structures. Shetty et al. [24] described a good indication for SITLLR in all the patients with well-localized lesions, whereas other authors focused on the importance of solitary tumors located on the anterolateral segments of the liver [25, 26, 28] or in the left liver lobe [21, 23] because the liver suspensory ligament helps for surgical-site exposure, and parenchymal division line remains in the same axis of the port site and instrumentations. Still, the specific technical problem created by the single caccess should not be underestimated, relying mainly on difficulties of exposure and on surgeon's and instruments' positions. Such ergonomic difficulties could lead to inadequate sections planes that could be a major concern for oncological indications, requiring safe but economic margins resection. Furthermore, other steps of SITLLR technique are limiting factors, like the frequent changes of the instruments for the surgeon's dominant hand and the use of IOUS through the single umbilical access. We agree with other authors [24] to consider limiting factors for SITLLR, patients with vascular involvement, extrahepatic disease, contraindication to laparoscopy, malignant lesions greater than 5 cm because the incision required to extract the specimen itself defeats the purpose of SITL. Other challenges in SITLLR remain technical difficulties related to massive liver dissection, access to the hilum for eventual Pringle maneuver, insertion of ultrasound probe through the single access, frequent alternation and adjustment of instruments, shifted division line, restriction by the length of the laparoscopic instrument, inappropriate placement of the drain, and control of bleeding during parenchymal dissection. Hence insertion of one or more trocars [22, 24] or conversion to open surgery [24] becomes strongly recommended. The following question relies on the identification of eventual intraoperative and early postoperative benefits of SITLLR as compared with multiport LLR. Regarding the results of LLR, even in the absence of randomized studies, several reports clearly indicated nowadays the advantages of this technique as compared with open liver resection, including less intraoperative blood losses, reduced requirements for blood transfusions [36–42], eventually associated with reduced postoperative complications rates and shorter hospital stays [38–40, 42, 43]. Short-term benefits of LLR, as compared with classical open liver resections, were strikingly observed in treatment of hepatocellular carcinoma in cirrhotic patients; in these cases, the avoidance of a subcostal incision interrupting venous portocaval shunts could be critical. Potential benefits of SITL, out of cosmetic outcomes, could be the postoperative pain, which has been considered one of the major outcome measures in the prospective randomized studies [44, 45], but a final sentence remains of concern. We achieved a nonadditional use of pain medication used for LLR in this initial experience as reported [21–23, 26], allowing the patients to be discharged before the postoperative day 5. In conclusion, at this time SITLLR remains a challenging procedure. Feasibility has been reserved for highly selected cases, but it has to be performed by formed surgeons in hepatobiliary and laparoscopic surgery with skills in general SITL. Apart from its cosmetic benefits, the future of this technique will be dependent on the confirmation of significant results if compared with multiport LLR and, then, of objective advantages, such as a reduction of the global operative stress and/or an improvement of postoperative outcomes.

## Figures and Tables

**Table 1:** Literature review for series of more than 5 cases.

Authors	Port	Instruments	Scope	Pathologies	Cases (*n*)	BMI (Kg/m^2^)^∗^	Operative time (min)^∗^	Conversion (%)	Blood loss (mL)^∗^	Final scar (cm)^∗^	Hospital stay (days)^∗^
Shetty et al. [24]	Gloveport (Sejong Medical)	Straight	5 mm Flexible	Malignant	24	NA	205^#^	8.3^+^ 16.6^@^	500^#^	5	8.5^#^
Cipriani et al. [23]	Triport (ACS), Quadriport (Olympus)	Straight	5 mm Flexible	Benign and Malignant	14	24.3	187	0^+@^	214	NA	5^#^
Zhao et al. [22]	Triport (ACS), 5-5-5 mm trocars	Straight and Articulating	5 mm Rigid and Flexible	Benign and Malignant	12	26.3	80.4	16.7^+^ 0^@^	45	2.5	4.3
Aikawa et al. [25]	SILS port (Covidien)	Straight	5 mm Flexible	Benign and Malignant	8	NA	148	0^+@^	2	3	6.2
Pan et al. [28]	10 mm and 5 mm trocars	Straight	10 mm	Benign and Malignant	8	26.2	89.7	0^+@^	64.3	2.5	3.7
Tan et al. [26]	various	Straight and Articulating	5 mm Flexible	Benign and Malignant	7	NA	142^#^	NA^+^ 0^@^	200^#^	NA	3^#^
Gaujoux et al. [21]	Gelport (Applied)	Straight	10 mm Flexible	Benign and Malignant	5	27.1	107	0^+@^	39	5	2
Cai et al. [27]	Triport (ASC)	Straight	10 mm Rigid	Benign	5	25.8^#^	87.3^#^	0^+@^	NA	NA	4.6^#^

^∗^Mean.

^#^Median.

^+^Additional trocar.

^@^Open surgery.

NA: Not available.

**Table 2:** Patients' characteristics.

Patients	Age	Sex	BMI	Indication	Intervention
	(years)		(Kg/m^2^)	(liver segment)	
1	53	F	20.8	Biliary cyst	Cyst unroofing
				(4, 7, 8)	
2	59	F	30.2	Biliary cyst	Cyst unroofing
				(3, 4)	
3	46	F	20.5	Biliary cyst	Cyst unroofing
				(4, 5, 6, 7)	
4	24	F	24.4	Hydatid cyst	Left lobectomy
				(2, 3)	
5	26	F	20.6	Hydatid cyst (7)	Pericystectomy
6	65	F	23	Colorectal metastasis (8)	Wedge resection

**Table 3:** Operative and postoperative outcomes.

Patients	Total operative time	Laparoscopic time	Blood loss	Final scar length	Length of stay	Followup
	(min)	(min)	(mL)	(mm)	(days)	(months)
1	90	81	50	14	3	25
2	89	71	40	14	3	13
3	138	115	400	16	4	3
4	114	96	350	20	5	9
5	185	160	500	16	5	7
6	158	140	200	15	4	6
MEDIAN	126	105.5	275	15	4	8
